# Proton Transfer in Molten Lithium Carbonate: Mechanism and Kinetics by Density Functional Theory Calculations

**DOI:** 10.1038/s41598-017-07726-3

**Published:** 2017-08-07

**Authors:** Xueling Lei, Kevin Huang, Changyong Qin

**Affiliations:** 1 Department of Physics, Jiangxi Normal University, Nanchang, Jiangxi 330022 China; 2 Department of Biology, Chemistry and Environmental Health Science, Benedict College, Columbia, South Carolina 29204 USA; 3 Department of Mechanical Engineering, University of South Carolina, Columbia, South Carolina 29207 USA

## Abstract

Using static and dynamic density functional theory (DFT) methods with a cluster model of [(Li_2_CO_3_)_8_H]^+^, the mechanism and kinetics of proton transfer in lithium molten carbonate (MC) were investigated. The migration of proton prefers an inter-carbonate pathway with an energy barrier of 8.0 kcal/mol at the B3LYP/6-31 G(d,p) level, which is in good agreement with the value of 7.6 kcal/mol and 7.5 kcal/mol from experiment and FPMD simulation, respectively. At transition state (TS), a linkage of O–H–O involving O 2p and H 1 s orbitals is formed between two carbonate ions. The calculated trajectory of H indicates that proton has a good mobility in MC, oxygen can rotate around carbon to facilitate the proton migration, while the movement of carbon is very limited. Small variations on geometry and atomic charge were detected on the carbonate ions, implying that the proton migration is a synergetic process and the whole carbonate structure is actively involved. Overall, the calculated results indicate that MC exhibits a low energy barrier for proton conduction in IT-SOFCs.

## Introduction

Solid oxide fuel cells (SOFCs) are of growing interest as clean energy source due to the advantages they provide, including high efficiency, low emission and fuel flexibility^1–8^. Both oxygen-ion (o-SOFCs)^1–4^ and proton conductor (p-SOFCs)^5–8^ based electrolytes have been extensively examined. The o-SOFCs normally operate at high temperatures of 700–1000 °C, causing severe issues like thermo-mechanical compatibility and chemical stability, and also leading to high fabrication cost. For p-SOFCs, the operating temperature is much lower (400–600 °C) using the perovskite of BaCeO_3_ (BC) and its doped derivatives as electrolytes^9–11^, making them promising candidates for commercial power generators. However, the cerates react badly with CO_2_ and become dysfunctional. Despite many efforts to improve the performance of BC-based electrolytes, BaZrO_3_ (BZ) and its derivatives^12–15^ are considered as alternatives. The BZ-based electrolytes are chemically stable, but challenged by low conductivity at the desired temperature range. To achieve the commercial feasibility, durable electrolytes with high conductivity at 400–600 °C is necessary.

Yttrium-doped barium zirconate (BZY) is one of the promising proton conducting electrolytes for intermediate temperatures (IT) SOFCs due to its excellent chemical stability and good bulk proton conductivity. However, its refractory nature makes itself very difficult to sinter and hard to achieve high density structure. More recently, Huang *et al*.^16, 17^ reported that densification of BZY can be promoted by adding molten carbonate (MC) as a sintering aid. MC was filled into the pores of BZY first by infiltration method. Interestingly, not only high density structure of BZY was observed, but also high ionic conductivity of 0.33 and 0.38 S/cm at 600 °C in 3% H_2_O-air and 3% H_2_O-H_2_, respectively. The contribution from proton transfer to the ionic conductivity was found to be about 55% and the MC phase had played a significant role in the proton conduction. The conduction mechanism was mainly proposed by the equation below.

1 $${{\rm{H}}}_{2}{{\rm{O}}}_{(g)}+{V}_{{\rm{O}}}^{\bullet \bullet }+{({{\rm{C}}{\rm{O}}}_{3})}_{{{\rm{C}}{\rm{O}}}_{3}}^{\times }={({\rm{O}}{\rm{H}})}_{{\rm{O}}}^{\bullet }+{({{\rm{H}}{\rm{C}}{\rm{O}}}_{3})}_{{{\rm{C}}{\rm{O}}}_{3}}^{\bullet }$$

This enhanced conductivity implies that the MC phase in BZY can provide extra channels for proton migration. Similar hybrid systems of MC with BZY, BCZY^18, 19^, and even oxygen-ion conductor^20–24^ as IT-SOFC electrolyte have been previously reported by other groups and largely enhanced cell performance was observed. Such composite electrolytes are easy to fabricate with low cost, which will open a door for developing novel electrolytes for IT-SOFCs. In addition, it is important to point out that the contribution from hydroxide ion (OH^−^) to the proton conductivity was not considered here, while this was reported to be noticeable in ref. 25. In fact, the effect of water on the proton conductivity was already examined independently by two different groups^17, 19^. Both reported that the conductivity was increased by only 6–8% when water partial pressure changes from 0 atm to 0.3 atm in 5% H_2_ Ar or N_2_. This increase was explained due to the reaction of water with the surface defect (oxygen vacancy) and carbonate to produce HCO_3_^−^ and OH^−^ as proton carriers. Therefore, it is fairly reasonable to think the contribution of HCO_3_^−^ to the proton conductivity is much larger than that of OH^−^. The role of OH^−^ is not examined in the current study, but should be considered in the future.

In bulk perovskite oxides, proton transfer occurs through hopping between adjacent oxygen ions at normal lattice sites via a Grotthuss-type mechanism^26^. Further, experimental and computational data indicate that the proton transfer involves two steps: rotational diffusion around an oxygen ion and transfer diffusion toward a neighboring oxide ion^27–32^. For proton conduction in BZY/MC as described in Eqn (1), the protons produced by surface defect reactions were transferred to the neighboring carbonate-ions (CO_3_^2−^) at the BZY/MC interface to form HCO_3_^−^. They will then be transported inside of the MC phase. However, such assumption has not been verified in any form yet. Previously, we have reported a static DFT study of the proton transfer in the crystal structure of lithium carbonate^33^. The calculated energy barrier was 0.34 eV along the direction of [001]. However, the experiments in refs 16 and 17 have shown that the MC phase only have large contributions to the ionic conductivity when it is melt at higher temperature than 450 °C. This implies that the results in ref. 33 are not enough for understanding the proton conduction mechanism in molten carbonate. In the current study, two significant improvements have been made. One is to use a disordered cluster model to represent the MC phase, which is close to the real situation and should produce more reliable results. The second is to include the effect of finite temperature using the first-principles molecular dynamics (FPMD) method.

In particular, the cluster of (Li_2_CO_3_)_8_ was used to represent the MC phase and those calculations were performed in two steps. In the first step, all gas phase geometries of [(Li_2_CO_3_)_8_H]^+^ cluster were optimized at the B3LYP^34, 35^/6–31 G(d,p)^36–39^ level using the Gaussian 09 program^40^. All stationary points on the potential energy surface (PES) were then verified by calculated vibrational frequencies at the same theoretical level. Therefore, the reactant and product structures are truly local minima while each transition state at the first order saddle point is on the PES. For such a large molecular system, locating a transition state is time consuming and challenging. To quickly achieve this, we performed a geometry scan first, then used the structure with the highest energy for TS search, and finally verified the reaction pathways using the intrinsic reaction coordinate (IRC) calculations. In the second step, we applied the VASP code (version 5.3.5)^41, 42^ to perform first-principles molecular dynamics (FPMD) calculations. The optimized structure of [(Li_2_CO_3_)_8_H]^+^ from step one is used as the starting geometry. All FPMD simulations were conducted with the NVT ensemble. PAW-PBE potentials^43–45^ were used for H (ultrasoft), Li (s1p0), C (s2p2), and O (s2p4) with the energy cutoff of 500 eV. A Verlet algorithm was integrated with Newton’s equations of motion at a time step of 0.1 fs for a total simulation time of 3 ps, *i*.*e*., 30000 time steps in total. The frequency of the temperature oscillations was controlled by the Nosé mass during the simulations. Additional, a 1 × 1 × 1 k-point mesh was used at the Γ-point.

The proton transfer process is local and the reaction is usually at high temperature in a molten carbonate salt, both of which will bring detriments to the results from static DFT calculations in the gas phase using a limited size of cluster model. For example, the calculated energy barrier includes contributions from not only the proton migration itself, but also the variation of the cluster geometry. Therefore, there are possible uncertainties in the calculated energetics. To verify and correct such possible errors, we have re-examined the energies from local structures extracted from the cluster and the revised values showed large improvements and better consistency with current FPMD results and previous experimental results^17^. In addition, long range interactions in the molecular system were estimated by CAM-B3LYP^46^, but no significant difference was observed.

## Results and Discussion

### DFT results of proton migration in the (Li_2_CO_3_)_8_ cluster

#### Intra-carbonate migration of proton

**Figure 1:**
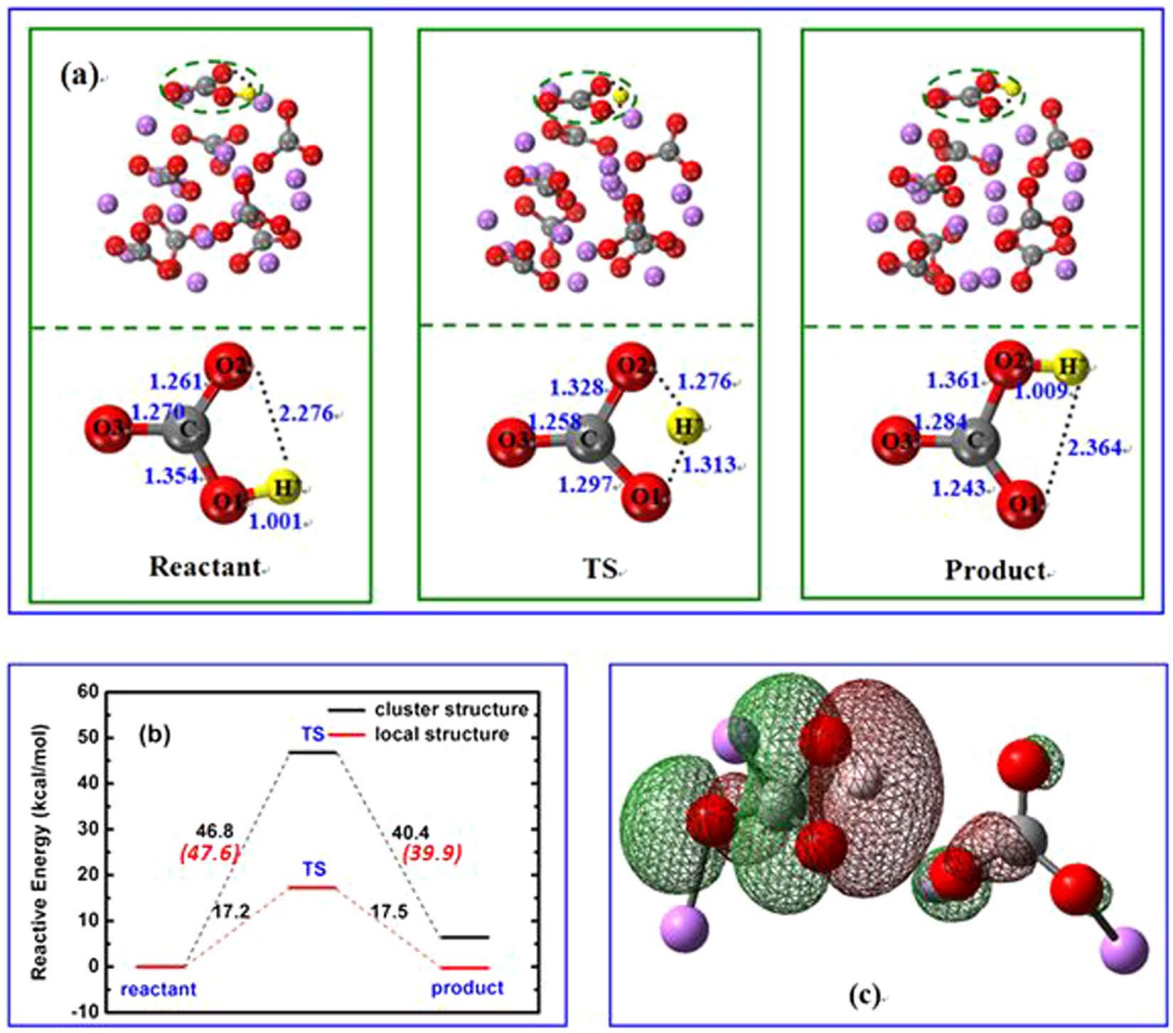
(**a**) Structures of reactant, transition state (TS) and product, the upper panel is the whole cluster, while local structure only for the lower panel; (**b**) Relative energy for proton migration inside of a carbonate ion calculated by the whole cluster and local structure, respectively; (**c**) Selected molecular orbital in TS for bonding between proton and carbonate. The proton, carbon, oxygen and lithium atoms are shown as yellow, gray, red, and purple respectively. (Distance in Å and relative energy in kcal/mol.).

For the migration of proton in MC, both intra and inter-carbonate pathways are possible and they will be discussed separately. As reported in ref. 33, the cluster of (Li_2_CO_3_)_8_ is fairly reliable to represent the disordered molten carbonate with affordable computing time. Figure 1 shows the optimized structures of reactant, transition state (TS), product together with the relative energy for proton migration inside of a carbonate ion in the cluster of (Li_2_CO_3_)_8_. At the beginning, the proton (H^+^) is connected to O1 with the O1-H^+^ distance of 1.001 Å. It then starts moving toward O2, leading to a TS. At TS, the only imaginary frequency is *i*1851 cm^−1^, corresponding to the shifting of H^+^ from O1 to O2. From reactant to TS, the O1-H^+^ bond is stretched to 1.313 Å, while O2-H^+^ shortened to 1.276 Å. At the same time, C-O1 is shortened from 1.354 Å to 1.297 Å, while C-O2 elongated from 1.261 Å to 1.328 Å. It is also noticeable that the O1-C-O2 bond angles is bent to 106.6°, which brings O1 and O2 closer and will facilitate the proton migration. The Mulliken population analysis shows a loss of 0.02 e for O1 from reactant to TS, gain of 0.05 e for O2 and loss of 0.04 e for carbon. All results indicate that the proton migration is a concerted process and involves 2p orbitals of O1-C-O2. Figure 1(c) displays the main bonding orbital between proton with O1 and O2 at TS. It is very clear that the 2p lone pairs of O1 and O2 combine with H 1 s, forming a bent O1-H-O2 linkage. The cleavage of O1-H^+^ and formation of O2-H^+^ is reversible as verified by the IRC calculations.

The calculated energy barrier for proton migration from O1 to O2 is 46.8 kcal/mol and 40.4 kcal/mol for the reverse process. The energy difference between the initial and final state is 6.4 kcal/mol. With long range corrections by CAM-B3LYP, the barrier is 47.6 kcal/mol and 39.9 kcal/mol, respectively, showing negligible contribution from long range interactions in the system. However, the value is only 20.5 kcal/mol in ref. 33, which was calculated based on a single molecule of HCO_3_^−^. To eliminate possible uncertainty in the calculated energy barrier as discussed before, we have extracted a small HCO_3_^−^ cluster out of the whole structure and re-calculated the energies. The energy barrier is decreased to 17.2 kcal/mol and 17.5 kcal/mol for the reverse step. The energy change for the migration process is reduced to 0.3 kcal/mol. Both imply that the contribution from geometry variation in the cluster other than proton migration itself is significant and the results from such calculations should be carefully evaluated and verified.

#### Inter-carbonate migration of proton

**Figure 2:**
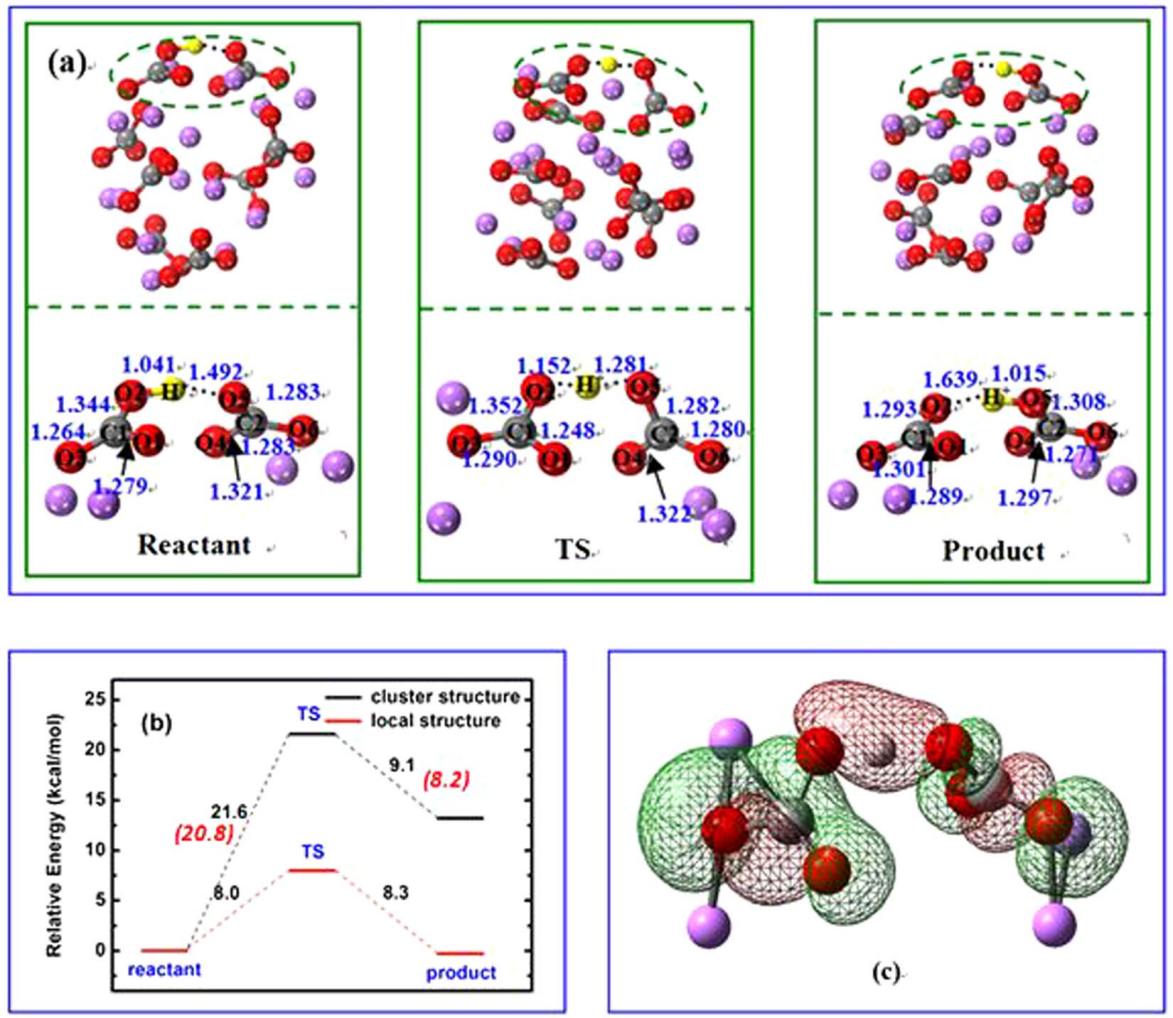
(**a**) Structures of reactant, transition state (TS), and product, the upper panel is cluster structure and the lower panel is local structure; (**b**) Relative energy for proton migration for inter-carbonate in the (Li_2_CO_3_)_8_ cluster and local structure, respectively; (**c**) Selected molecular orbital in TS for bonding between proton and carbonate. The proton, carbon, oxygen and lithium atoms are shown as yellow, gray, red, and purple respectively. (Distance in Å; Relative energy in kcal/mol.).

Figure 2 shows the optimized structures of reactant, TS, product as well as the relative energy for proton migration through an inter-carbonate pathway in the cluster of (Li_2_CO_3_)_8_. Unlike the intra-carbonate process, this pathway involves two neighboring carbonates. As shown in Fig. 2(a), a TS structure for the migration of H^+^ between O2 and O5 was located on the PES. At TS, the only imaginary frequency is *i*387 cm^−1^, corresponding to the migration of H^+^ from O2 to O5. At the beginning, the O2-H^+^ distance is 1.041 Å, similar to O1-H^+^ in Fig. 1(a). However, it is only 1.492 Å for O5-H^+^, which is 0.784 Å shorter than O2-H^+^ in Fig. 1(a), implying that the inter-carbonate pathway is possibly favored by structure. At TS, a linear O2-H-O5 linkage is formed between two carbonate ions with the O2-H^+^ and O5-H^+^ distance of 1.152 Å and 1.281 Å, respectively. Figure 2(c) shows large overlap between 2p of O2 and O5, and 1 s of H. The Mulliken population analysis indicates a gain of 0.10 e for O2, loss of 0.02 e for O5, and gain of 0.03 e for C1 from reactant to TS. A similar trend is observed for the reverse path from product to TS. Although the absolute values of charge are not very reliable, the small variations of electron density on the atoms where the proton migration occurs indicate that this is a synergetic process between proton and the two carbonate ions.

The calculated energy barrier is 21.6 kcal/mol from reactant to TS, while only 9.1 kcal/mol from product back to TS. It becomes 20.8 kcal/mol and 8.2 kcal/mol after the long range correction by CAM-B3LYP, respectively. The large energy difference between reactant and product (12.5 kcal/mol) implies the fact that the cluster of product from IRC might be a local minimum on PES, i.e. an intermediate structure. A similar phenomenon was observed in ref. 33, where a very small activation energy of 1.8 kcal/mol was reported for the proton transfer between two carbonate ions. Using an extracted cluster Li_2_CO_3_-H^+^-Li_2_CO_3_ as shown in Fig. 2(a), the energy barrier is reduced to 8.0 kcal/mol and 8.3 kcal/mol, respectively. This again confirms that the gas phase calculation may overestimate the activation energy for a molten salt system using a simple cluster model. From the current study, it is found that the calculated energy barriers will have to be checked and verified by other methods, which will be discussed in more details. Comparison of the calculated energy barriers suggests that the proton migration proceed through inter-carbonate rather than intra-carbonate in the lithium molten carbonate, which agrees with the results from the geometry and charge analysis.

### FPMD evidences of proton migration in the (Li_2_CO_3_)_8_ cluster

#### Trajectories of the proton, carbon, and oxygen atoms

**Figure 3:**
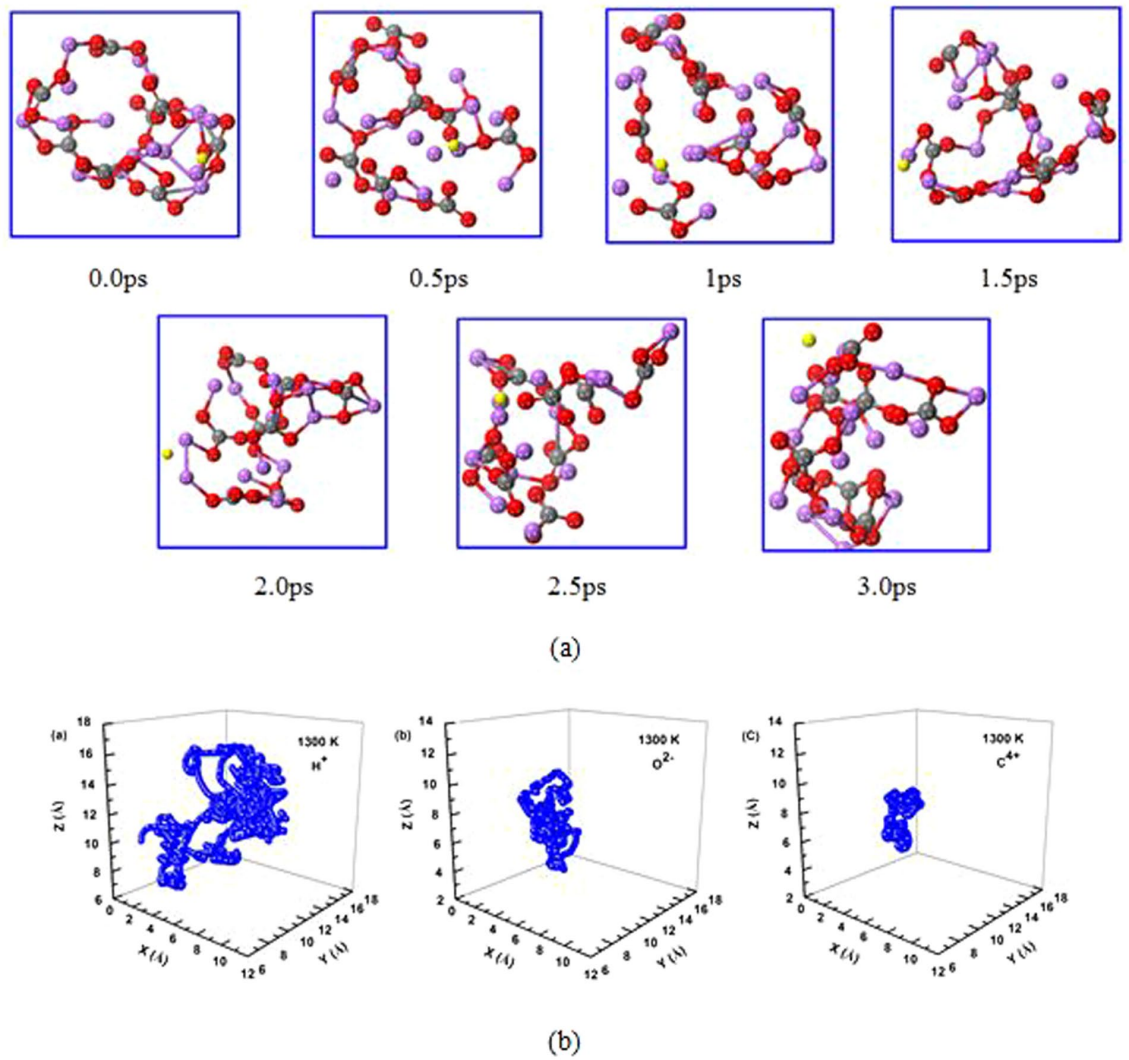
(**a**) Snap-shots for proton transfer in the cluster of (Li_2_CO_3_)_8_ simulated at 1300 K for 3 ps. The yellow ball represents the proton in motion. (**b**) Trajectory of proton, carbon, and oxygen ion in the cluster of (Li_2_CO_3_)_8_ simulated at 1300 K for 3 ps.

To account for the effects of temperature on the proton migration in the lithium molten carbonate, we carried out first-principles molecular dynamics calculations. Figure 3 shows a series of the snapshots of proton position at 1300 K in a time interval of 0.5 ps and the corresponding trajectory of proton, carbon and oxygen ion in the (Li_2_CO_3_)_8_ clusters simulated at 1300 K for 3 ps. The proton position in each snapshot has experienced noticeable change with the time, indicating a fast proton diffusion process. The trajectory of proton is largely dispersed while the trajectory of carbon is quite localized, indicating that the proton migration in the (Li_2_CO_3_)_8_ cluster is very fast with large displacement, while the carbon atoms only vibrate around their original sites with very limited displacement. It should also be noted that the trajectory of the oxygen atom is more dispersed than that of carbon, implying that the oxygen atoms can rotate and adjust their positions during the proton migration between the carbonate ions in the cluster of (Li_2_CO_3_)_8_.

#### Mean square displacement and Arrhenius plot

**Figure 4:**
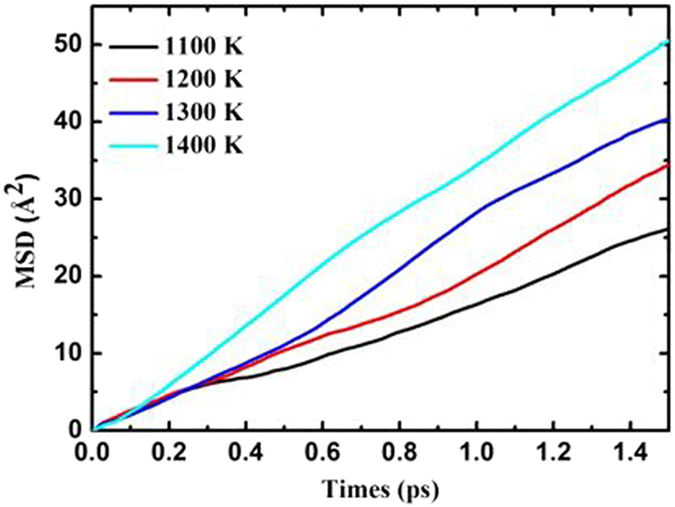
MSD for proton transfer in the cluster of (Li_2_CO_3_)_8_ simulated at 1100 K, 1200 K, 1300 K, and 1400 K for 3 ps.

The mean square displacement (MSD) is determined by the ensemble average:

2 $$MSD(t)=\langle\delta {r}^{2}(t)\rangle=\frac{1}{N}\frac{1}{n}\sum _{i=0}^{n}\sum _{m=0}^{N}{[{r}_{m}(t+{t}_{0i})-{r}_{m}({t}_{0i})]}^{2}$$

where *N* is the number of ions in the system, *n* is the number of time origins, *t* is the time, and *t*_0*i*_ is the initial time step originating at time *i* ^47, 48^. In the current work, at least three FPMD runs were conducted for one structure at each temperature in order to obtain an averaged MSD for better reliability and accuracy. Figure 4 shows the averaged mean square displacement for proton migration in the (Li_2_CO_3_)_8_ clusters simulated at the temperature of 1100 K, 1200 K, 1300 K and 1400 K for 3 ps (here considering the melt point of lithium carbonate is about 996 K). Clearly, the MSD of proton increases linearly with time, indicating a fast proton migration in the (Li_2_CO_3_)_8_ clusters. Moreover, the slope of MSD increases as the temperature increases, indicating that the proton has higher ability of diffusion at increased temperature.

The diffusion coefficient is calculated by fitting the slope of the MSD vs. time:

3 $$D=\mathop{\mathrm{lim}}\limits_{t\to \infty }\frac{MSD(t)}{6t}$$

Then, the Arrhenius plot can be obtained from the diffusion coefficient via Arrhenius relationship:

4 $$D(T)={D}_{0}(T)\exp (-\frac{{E}_{a}}{{k}_{B}T})$$

**Figure 5:**
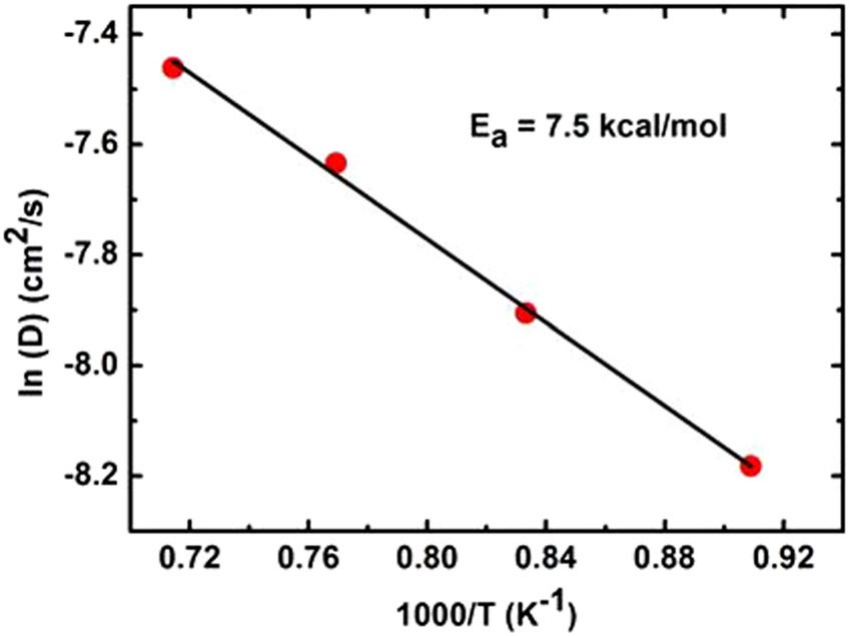
Arrhenius plot for proton transfer in the cluster of (Li_2_CO_3_)_8_.

Herein, ${D}_{0}$ and ${E}_{a}$ are the pre-exponential factor and the diffusion barrier, respectively. ${k}_{B}$ is the Boltzmann constant and T is the absolute temperature. By fitting to the Arrhenius relationship (Eq. 4) over the considered temperature range, the pre-exponential factor and the effective activation energy can be obtained. Figure 5 shows the Arrhenius plot for proton migration in the cluster of (Li_2_CO_3_)_8_. From the slope of the straight line, the activation energy is calculated to be 7.5 kcal/mol, which is in good agreement with the energy barrier of 8.0 kcal/mol for the proton migration between carbonate ions in the cluster of (Li_2_CO_3_)_8_. The trajectory analysis and the activation energy strongly support that the proton transfer prefers the inter-carbonate pathway and suggest that static DFT methods with a limited size cluster model in the gas phase has uncertainties in describing the state of molten carbonate and that further refinement on calculated energies with extracted local structure is necessary. Such treatment can also be applied in treating similar molecular systems in future.

### Mechanism of proton transfer in MC

Due to the fact that the mechanism of proton migration in MC is complicated, both static DFT and FPMD calculations at finite temperatures have been performed in the current study. The results from DFT calculations in the gas phase have provided detailed information on structural evolution, charge transfer and energetics, while trajectories, MSD at different finite temperatures and activation energy by Arrhenius plot were obtained from FPMD. All theoretical evidences from multiple perspectives have supported that the inter-carbonate pathway is dominant for the proton migration in molten carbonate. However, the intra-carbonate pathway is still possible to serve as an intermediate step in the whole process. In addition, the combination of the static and dynamic DFT methods in this study has shown great advantages in treating such ionic liquid systems.

**Figure 6:**
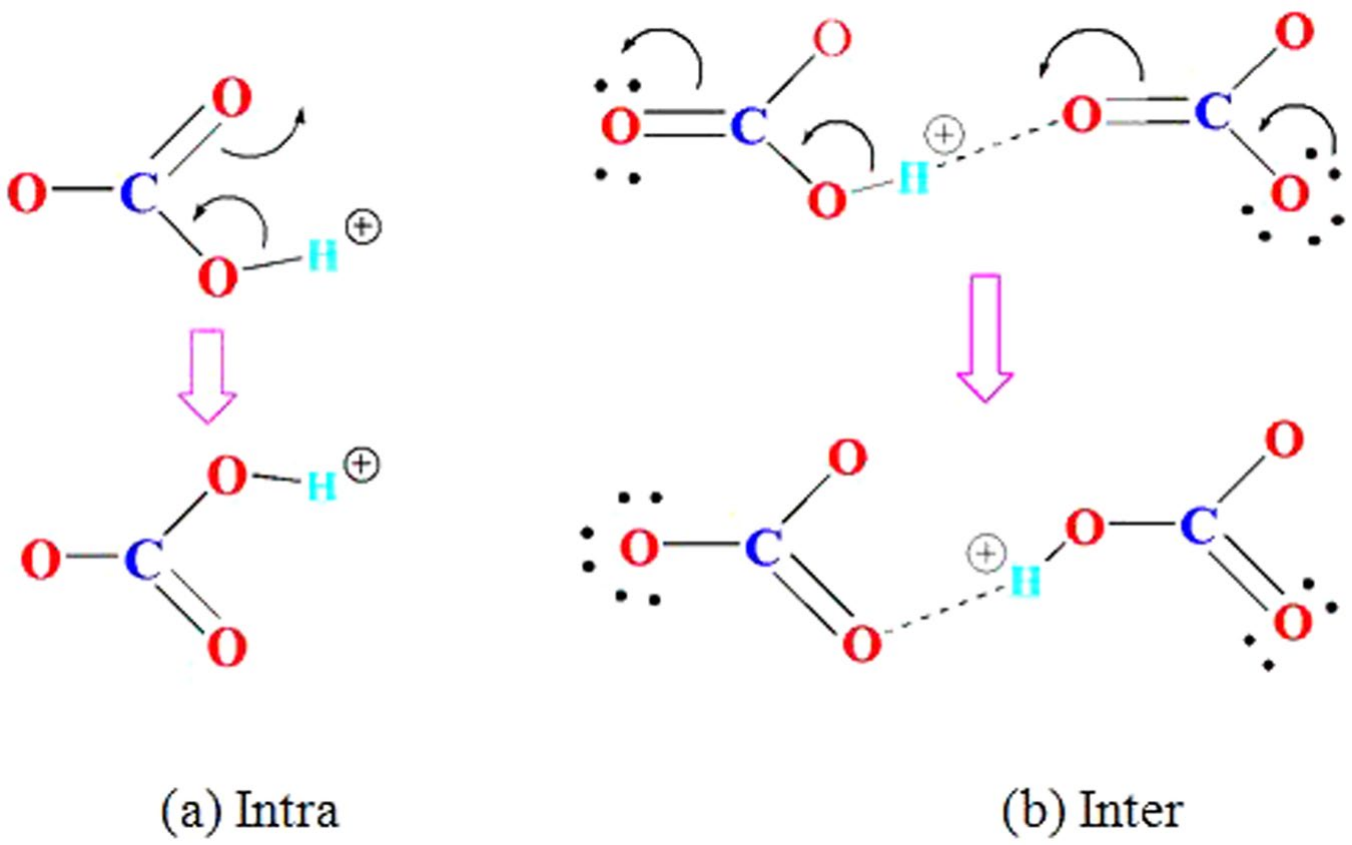
Lewis Diagram for the Intra- (**a**) and Inter-Carbonate (**b**) Migration of Proton.

It is interesting to note that the degree of geometry change of the carbonate ions at TS is slightly different from the intra to inter-carbonate pathway. In Fig. 1(a), the changes of the O3 charge and O3-C bond distance are significantly smaller than those for other atoms and bonds in the carbonate ion, indicating O3 is not actively participated in the proton migration process. However, both O1-C and O2-C bonds change significantly during the process. One bond increases and the other will decrease accordingly. The O1-C-O2 bond is bent to 106.6° to accommodate the proton migration. This is consistent with the Lewis diagram in Fig. 6(a). With respect to the inter-carbonate path, all atoms in both carbonate ions have experienced some small changes in atomic charge and bond distance, showing they are all actively involved in the process. The O-C-O bond angles remain almost unchanged (~120°) in the whole process. The bond forming and breaking process can be described by the Lewis diagram in Fig. 6(b). Theoretically, if a reaction experiences more electron delocalization and less geometry distortion at TS, it should have a lower energy barrier. From this chemical aspect, the inter-carbonate path should be more favored by reaction kinetics.

For the energy barriers from static DFT calculations, they are obviously overestimated. This uncertainty is probably due to the structural variation of the ionic cluster, especially the part not involved in the proton migration in the optimization process. The value of energy barrier itself is small, but the total energy of the cluster is large. To correct the errors, we have extracted the small local structure out of the whole cluster, which allows us to catch the energy change due to the proton migration and ignores the effect on the energy from the ions not involved in the process. The energy barrier was reduced from 46.8 to 17.5 kcal/mol for the intra-carbonate path and from 20.8 to 8.0 kcal/mol for the inter-carbonate one, respectively. Such large changes imply the challenges in treating such an ionic liquid of molten salt state using gas phase DFT and cluster models. The correction method used here has been very effective and is easy to apply, and further verifications will be carried out in the future. The final energy barrier from DFT is 8.0 kcal/mol, which is in a good agreement with the activation energy of 7.5 kcal/mol from FPMD simulations. The activation energy was measured to be 7.6 kcal/mol using a BZY/MC composition electrolyte at 600 °C and MC was considered as the main conducting media for protons^17^. Based on those values, we can conclude that the energy barrier for the migration of proton in molten lithium carbonate salt is in the range of 7.5–8.0 kcal/mol and excellent consistency between DFT modeling and experimental measurement was observed.

## Conclusions

The pathway and energy barrier of proton migration in the lithium molten carbonate are predicted by static and dynamic density functional theory calculations. The results show that the proton migration prefers the inter-carbonate to the intra-carbonate pathway. The migration energy barrier of 8.0 kcal/mol for local structure extracted from the (Li_2_CO_3_)_8_ cluster is consistent with the 7.5 kcal/mol obtained from *ab initio* molecular dynamic simulations. Also, this result is excellent in line with the experimental value of 7.6 kcal/mol observed in a MC/BZY composition at 600 °C. The analyses of atomic charges, bond distances, bond angles, vibrational frequencies, and molecular orbitals all indicate that the proton migration in the lithium molten carbonate is a synergetic process. The trajectories of proton, carbon and oxygen suggest that the oxygen atoms can adjust their positions during the proton migration between the carbonate ions in the cluster of (Li_2_CO_3_)_8_. The MSDs of proton simulations at temperatures of 1100 K, 1200 K, 1300 K, and 1400 K for 3 ps feature a linear increase with time, implying a fast proton migration in the lithium molten carbonate. Moreover, the mechanism of proton migration can be explained by the Lewis electron dot diagram. Overall, the calculated results have an excellent agreement with our previous experimental results and indicate that molten carbonate can provide a low energy barrier channel for proton conduction in IT-SOFCs. The kinetics and mechanism of such process are well elucidated by DFT calculations. In addition, our calculations indicate that the combination of the static and dynamic DFT methods is of great advantages in treating such ionic liquid systems and can give reliable results.
